# Molecular Simulation-Based Investigation of Highly Potent Natural Products to Abrogate Formation of the nsp10–nsp16 Complex of SARS-CoV-2

**DOI:** 10.3390/biom11040573

**Published:** 2021-04-14

**Authors:** Anwar Mohammad, Eman Alshawaf, Sulaiman K. Marafie, Mohamed Abu-Farha, Fahd Al-Mulla, Jehad Abubaker

**Affiliations:** 1 Department of Biochemistry and Molecular Biology 1, Dasman Diabetes Institute, Dasman 15462, Kuwait; eman.alshawaf@dasmaninstitute.org (E.A.); sulaiman.marafie@dasmaninstitute.org (S.K.M.); mohamed.abufarha@dasmaninstitute.org (M.A.-F.); 2 Department of Genetics and Bioinformatics 2, Dasman Diabetes Institute, Dasman 15462, Kuwait; fahd.almulla@dasmaninstitute.org

**Keywords:** non-structural protein, SARS-CoV-2, natural product, molecular dynamic simulation, nsp10–nsp16 complex

## Abstract

The SARS-CoV-2 non-structural protein (nsp) nsp10–nsp16 complex is essential for the 2′-O-methylation of viral mRNA, a crucial step for evading the innate immune system, and it is an essential process in SARS-CoV-2 life cycle. Therefore, detecting molecules that can disrupt the nsp10–nsp16 interaction are prospective antiviral drugs. In this study, we screened the North African Natural Products database (NANPDB) for molecules that can interact with the nsp10 interface and disturb the nsp10–nsp16 complex formation. Following rigorous screening and validation steps, in addition to toxic side effects, drug interactions and a risk /benefit assessment, we identified four compounds (genkwanin-6-C-beta-glucopyranoside, paraliane diterpene, 4,5-di-p-trans-coumaroylquinic acid and citrinamide A) that showed the best binding affinity and most favourable interaction with nsp10 interface residues. To understand the conformational stability and dynamic features of nsp10 bound to the four selected compounds, we subjected each complex to 200 ns molecular dynamics simulations. We then calculated the free binding energies of compounds interacting with nsp10 structure using the molecular mechanics-generalised Born surface area (MMGBSA). Of the four compounds, genkwanin-6-C-beta-glucopyranoside demonstrated the most stable complex with nsp10, in addition to a tighter binding affinity of −37.4 ± 1.3 Kcal/mol. This potential to disrupt the nsp10–nsp16 interface interaction and inhibit it now sets the path for functional studies.

## 1. Introduction

The spread of severe acute respiratory syndrome coronavirus 2 (SARS-CoV-2) has caused the ongoing coronavirus disease 2019 (COVID-19) pandemic. Similar to the SARS-CoV and the Middle East respiratory syndrome (MERS-CoV), SARS-CoV-2 belongs to the Betacoronavirus family and is associated with symptoms of severe pneumonia and bronchiolitis [1,2,3,4,5]. Existing conditions, such as cardiovascular disease (CVD), diabetes and renal disease, influence the severity and outcomes of SARS-CoV-2 infection [6]. The SARS-CoV-2 genome consists of enveloped non-segmented positive single-stranded RNA molecules with the invariant gene order 5′−replicase-S-E-M-N−3′. The 5′–replicase complex is responsible for viral RNA replication and transcription [7,8,9]. This complex includes two large overlapping open reading frames (ORFs), where ORF1a encodes polyprotein pp1a and ORF1b encodes polyprotein pp1ab. Autoproteolytic cleavage of the polyproteins pp 1a/1ab results in 16 non-structural proteins (nsp1–16) [10,11,12]. The end products of the pp1a cleavage are involved in preparing the cell for infection and possess the RNA synthesis machinery, whereas pp1ab end products catalyse RNA replication and transcription [7,8,9]. Nsp10, a pp1a product that is highly conserved in coronaviruses, has been identified as an essential protein for viral replication through its formation of complexes with nsp14 and nsp16 [13,14,15,16,17].

Upon infection, the viral RNAs are identified through the TLR7-recognition pathway, thereby triggering interferon type I (IFN-I) to modulate the innate immune response [18,19,20]. Coronaviruses escape this immune system detection by employing their encoded methyltransferases (MTase) to mask their presence through mRNA capping [21,22,23], thereby protecting viral mRNA against exoribonucleases and promoting translation initiation [24,25]. Nsp16 is involved in 2′-O-methylation of the viral mRNA cap to form cap-1 and cap-2 structures, a crucial step in evasion of the host’s innate immune system [21]. In SARS-CoV-2, nsp16 interacts with nsp10 to stabilise the binding of S-adenosyl methionine (SAM) to the nsp16 pocket and extend its substrate RNA-binding groove to trigger the nsp16 2′-O-MTase activity [22,26]. Since mRNA cap methylation is an essential process in the SARS-CoV-2 life cycle, the proteins of the MTase complex are potential targets for the development of antiviral drugs.

In SARs-CoV studies, Almazán et al. demonstrated that genetic disruption of nsp16 leads to a ten-fold decrease in viral RNA synthesis [27]. Moreover, the introduction of a short peptide that disturbed the interaction between SARS-CoV nsp10 and nsp16 inhibited 2′-O-MTase activity, which impaired virus pathogenesis and virulence by suppressing its replication and enhancing early-stage IFN-1 production [28,29]. In a recent in silico study, a range of compounds was tested against the stimulatory factor of MTase-nsp10–nsp16 complex of SARS-CoV-2 [30]. Moreover, recent structural analysis studies presented the importance of 2′-O-MTase nsp16 of SARS-CoV-2 as a drug target [31,32]. Nsp10 residues 42–120 were shown to be the functional area required for stimulating nsp16 enzymatic activity [28]. By contrast, the conserved residues in nsp16 that are required to form the nsp10–nsp16 complex are scattered throughout a large region of the protein and are brought together in the interface by protein folding [29]. The interaction interface of nsp10 therefore appears to be an attractive site for the design of MTase inhibitory peptides and this also makes it favourable for the development of potential antiviral drugs. Since the nsp10–nsp16 complex is crucial for the survival of the SARS-CoV-2 virus, finding compounds that have the potential to disrupt this interaction would protect against SARS-CoV-2 infection [32].

For instance, computational methods such as virtual screening of medicinal databases, molecular dynamics simulations and free energy calculations are of great interest to understand the molecular mechanism of pathogenesis, repurposing the old drugs or to discover new lead compounds for the treatment of COVID-19 [33]. In the present study, we screened the NANPDB [34] resource drugs database for molecules that interact with the nsp10 interface and disturb the nsp10–nsp16 complex formation. The database comprises natural drugs from 617 source species and 146 families of plants, animals, bacteria and fungi from Northern and South Africa. It provides rich metadata, literature references and cross-references to major chemical databases that can be easily downloaded and constantly being updated to include data published on newly identified compounds in the Eastern African region [35]. Interestingly, this database includes compounds with anti-viral (HCV and HIV) activities, and others reported with anti-malarial anti-plasmodial properties. NANPDB offers a comprehensive dataset for the compounds including source species, the reported biological activity and the predicted drug-likeness properties.

Following rigorous virtual screening and validation steps, we identified four plant compounds with the best binding affinity and most favourable interaction with nsp10 interface residues. We further tested the conformational stability and dynamic features of nsp10 bound to the four selected compounds by subjecting each complex to 200 ns molecular dynamic (MD) simulations. We used the MMGBSA to extract free binding energies. Of the four compounds, genkwanin-6-C-beta-glucopyranoside demonstrated the most stable complex with nsp10, in addition to a tighter binding affinity (−37.4 ± 1.3 Kcal/mol), thereby setting the path for functional in vitro and in vivo studies.

## 2. Methods

### 2.1. nsp10–nsp16 Structure

Several SARS-CoV-2 nsp10-nsp16 structures had been deposited in the RCSB Protein Data Bank (PDB), by Rosas-Lemus et al. (PDB ID:6W61) [26], Krafcikova et al. (PDB ID:6YZ1) [36] and Viswanathan et al. (PDB ID: 6WKS) [22]. We utilised the first SARS-CoV-2 nsp10-nsp16 structure deposited in the PDB database (PDB ID:6W61) by Rosas-Lemus et al. [26] for docking natural products and the structural analysis. Moreover, the PDB structure 6W61 was validated against the X-ray crystal structure of nsp10-nsp16 by Krafcikova et al. (PDB ID:6YZ1) [36] using PyMOL [37]. Furthermore, to detect potential binding pocket based on the structure nsp10-nsp16, Protein Plus DoGSiteScorer [38], binding site detection was used to calculate the druggability score. The druggability score values range between zero and one, whereby the higher the score suggests the pocket is estimated to be more druggable. Residues involved in the nsp10-nsp16 binding interface were extracted from the nsp10–nsp16 complex using InterfaceResidues.py [39] in addition to PDBsum Prot-Prot analysis [40,41].

### 2.2. Virtual Screening of Compounds Interacting with nsp10

Northern and South African natural compounds were retrieved from NANPDB (Retrieved 20 December 2020 from https://african-compounds.org/nanpdb/) [34]. The data comprising 6482 compounds were downloaded in an SDF format, and the structure of each compound was prepared by compound washing, charges and minimisation. As mentioned above the druggability of the binding interface (nsp10-nsp16) was confirmed using DoGSiteScorer and virtual drug screening of the selected database was then processed with the Molecular Operating Environment (MOE v2016) [42], using the selected residues option to define the nsp10 interface residues. The compounds were limited to 10 conformations, and they were screened against the nsp10 interface using a triangle matcher and London dG as a scoring method. The selection criterion for choosing the best hits was based on docking scores and visualisation of the interaction with PyMOL [37] and VMD [43]. In total, 123 compounds fit the initial selection criterion.

To select the best active compounds that can interact with the nsp10 interface residues, the 123 compounds were subjected to pharmacokinetics and pharmacodynamics analysis using ADMET and pkCSM [44]. Validation from SwissADME [45] omitted 28 compounds. The remaining 95 compounds were further screened using an induced-fit docking protocol of MOE, which yielded 22 compounds with the highest binding affinities.

The final step was re-docking of the 22 compounds with nsp10. AutoDock Vina [46] was used to perform the induced fit docking (IFD) with 30 conformers while keeping the parameters at default settings. IFD modelling offers mutual conformation of the protein receptor to a ligand, thereby providing better accuracy than docking to a rigid target. The protein and compound structure were converted to .pdqt format, and the interaction interface residues were defined in the three-dimensional grid (x = −51.54 y = 78.58 z = 11.87) while the grid dimension was 30 × 30 × 30 with the exhaustiveness to set to 64 achieve high accuracy. The compounds were prepared for the docking run using root detection, charges, hydrogen and aromaticity criteria, and the top 10 were selected. In addition, Molinspiration Cheminformatics was used to test the bioactivity of the selected compounds [47]. The software predicts the molecular properties such as logP, number of H-bond donors and acceptors, and polar surface area in addition to the bioactivity score which is important for drug targets. This prediction validates the bioactivity of the selected best hits which further increases the reliability of the selected compounds for binding analysis with nsp10.

### 2.3. Molecular Dynamics Simulation of nsp10 Compound Complexes

The stability of each generated complex was checked by MD simulation performed on Amber20 [48] using the FF14SB force field [49]. The system solvation was performed in a rectangular water box with a TIP3P water molecule, and the system was neutralised by the addition of Na^+^ ions [50]. An energy minimisation protocol was used for the removal of the bad clashes in the system. The steepest descent algorithm [51] and the conjugate gradient algorithm were used for 6000 and 3000 cycles, respectively [52]. After heating up to 300 K, the system was equilibrated at a constant 1 atm pressure with weak restraint and then equilibrated without any restraint. Finally, the production step was run for 50 ns. The long-range electrostatic interaction was treated with a particle mesh Ewald algorithm [53] with a cut-off distance of 10.0 Å. The SHAKE algorithm was used to treat covalent bonds if they exits [54]. The production step of MD simulation was performed on PMEMD.CUDA, and trajectories were processed using the Amber20 CPPTRAJ package [55].

### 2.4. Binding Free Energy Calculation

The real binding energy calculations of the nsp10 bound to compounds were estimated using the MMGBSA approach. This method was the best approach used by different studies to estimate the real binding energy of different biological complexes, such as protein-ligand, protein-protein and protein-DNA/RNA complexes [56]. The MMGBSA.py [57] script was used to estimate the total binding free energy of the top ligand complexes. Each energy term, such as van der Waals (vdW), electrostatic, generalised Born (GB) and surface area (SA), were calculated as a part of the total binding energy.

The free energy was calculated by analysing 2500 MD trajectory structural frames of nsp10 in complex with the compound using the following equation [29].

(1) $$\Delta G_{bind}=\Delta G_{complex}-\left[\Delta G_{receptor}+\Delta G_{ligand}\right]$$

In this Equation (1), Δ*G*_bind_ represents the total binding free energy, while Δ*G*_complex_, Δ*G*_receptor_ and Δ*G*_ligand_ represent the free energy of the complex, the protein and the ligand, respectively. The following equation was then used to calculate a specific energy term that contributes to the whole free energy.

(2) $$G=G_{bond}+G_{ele}+G_{vdW}+G_{pol}+G_{npol}-TS$$

In Equation (2), the *G*_bond_ is the summation of the bond, angle and dihedral energies, whereas the *G*_ele_ and *G*_vdW_ are the electrostatic and van der Waals energies, respectively. The *G*_pol_ and *G*_npol_ are the polar and nonpolar contributions to the solvation energy, respectively, with G_pol_ calculated by solving the PB equation and *G*_npol_ estimated from the linear relationship with the surface accessible surface area (SASA). The configurational entropy (TS) is typically ignored because of the greater computational costs [58].

## 3. Results and Discussion

### 3.1. nsp10–nsp16 Structure and Interface and Cavity

The nsp10–nsp16 complex consists of 298 residues. The nsp16 structure contains the 2‘-O-MTase catalytic core, while nsp10 comprises 139 residues with two Zn binding sites. The positively charged nsp10 surface and the hydrophobic pocket interact with the negatively charged surface and hydrophobic core of nsp16, respectively, to form an interface that has been a drug target in SARS-CoV studies [28] and computational and isothermal titration studies SARS-CoV-2 studies [30,32,59]. Consequently, disrupting the nsp10–nsp16 interface could protect against viral infection.

**Figure 1:**
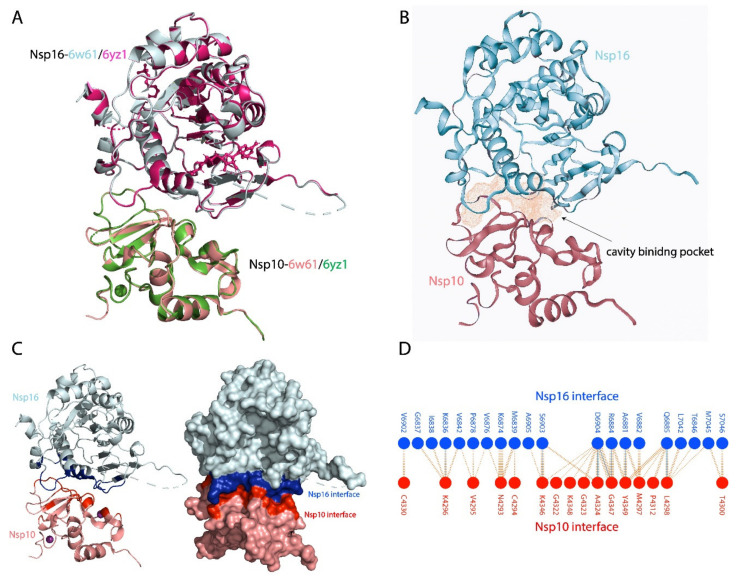
(**A**) Structural superposition of nsp10- 6W61 (salmon) with nsp10-6YZ1 and nsp16-6W61 (cyan) with nsp16-6WZ1 (magenta) with a root-mean-square deviation (RMSD) of 0.159 Å. (**B**) Cavity binding pocket of nsp10-nsp16 structure (6w61). (**C**) Showing the interface of the nsp10-nspa6 binding protein partners, with blue colour represents the nsp16 binding interface residues while the red colour represents the nsp10 binding residues. The right panel in the (**C**) shows the nsp10-nsp16 complex as surface. (**D**) Schematic of the bonding interactions between interface residues of nsp10 (red) and nsp16 (blue).

Some reports have characterised the structural features of the heterodimer protease complex (i.e., nsp10–nsp16 2′-O-MTase) in SARS-CoV-2 at the atomic level. In particular, these studies have targeted the conserved S-adenosyl-L-methionine (SAM)-binding pockets with anti-viral and anti-protease compounds [30,32]. In our study, we utilised the recently solved X-ray crystal structure of the SARS-CoV-2 nsp10–nsp16 complex (PDB ID:6W61) (Figure 1) to identify natural compounds from the NANPDB that can disrupt the nsp10–nsp16 interaction and abolish nsp16 activity. The structure was validated against the X-ray crystal structure of nsp10-nsp16 by Krafcikova et al. (PDB ID:6YZ1) [36] using PyMOL with a root-mean-square deviation (RMSD) of 0.159 Å between 6YZ1 and 6W61 structures (Figure 1A). Using Protein Plus DoGSiteScorer Binding site detection, the potential binding pocket based on the structure nsp10-nsp16 was detected (Figure 1B). The cavity depicted in Figure 1B presented the highest druggability score. 0.85 indicating the best region for drug binding. The nsp10 structure (residues 4272–4392 of pp1a) was extracted from the nsp10–nsp16 complex. In addition, the nsp10 residues positioned in the interaction interface were identified using a script InterfaceResidues.py and the protein structural analysis software PyMOL [37] (Figure 1B). PDBsum prot-prot analysis depicted interface residues on the secondary structure elements (Figure 1D) corroborate with the interface residues shown by Viswanathan et al. in their nsp10–nsp16 structural studies [22].

### 3.2. Virtual Drug Screening and Molecular Docking

**Figure 2:**
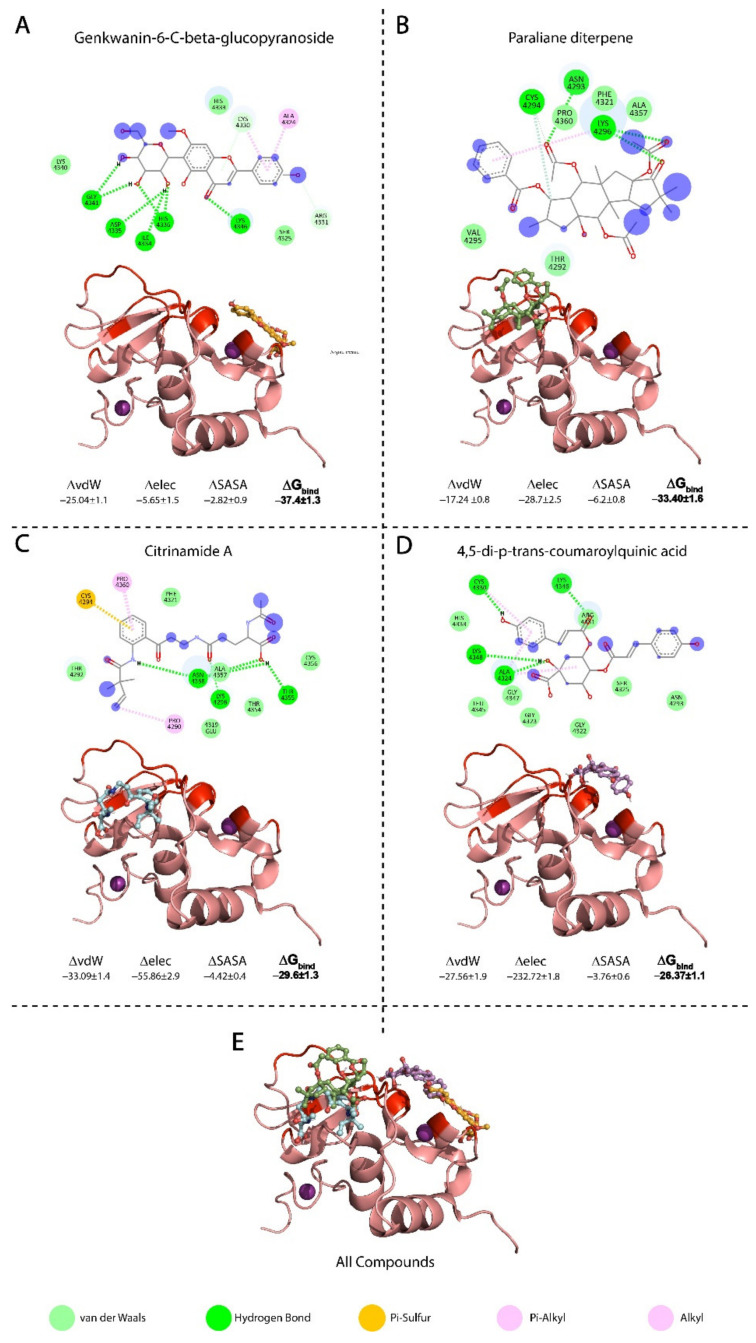
The four compounds (**A**) Genkwanin-6-C-beta-glucopyranoside-nsp10 complex, (**B**) Paraliane diterpene-nsp10 complex, (**C**) Citrinamide A, (**D**) 4,5-di-p-trans-coumaroylquinic acid (**E**) All four compounds. The 2D structured of natural products and the 3D nsp10 structure (salmon) interacting with the interface residue (red) interacting with the compounds. The free binding energies of compounds interacting with nsp10 structure was calculated using the Generalised-Boron surface area molecular mechanics (MMGBSA).

The search for a potential drug that interacts with the SARS-CoV-2 nsp10 interface involved a multi-step drug screening processes of the 6842 NANPDB resource drugs database. NANPDB is a diverse database of medicinal plants containing natural drugs from 617 source species from 146 families of plants, animals, bacteria and fungi from Northern and South Africa. The initial step comprised a screening of all the compounds in the databases using Molecular Operating Environment (MOE) [42], with a total of 123 compounds fitting the selection criteria. The 123 compound conformations were manually visualised and filtered based on their docking scores, where the criterion for interactions with the interface residues of nsp10 had a score range of −7.84 to −5.0 kcal/mol. Absorption, distribution, metabolism, excretion and toxicity (ADMET) [45] screening analysis of the 123 compounds eliminated 28 compounds, leaving a remainder of 95 compounds with the best fit. The remaining 95 compounds were screened against the nsp10 interface residues using an induced fit model (IFD) method, with scores ranging between −8.52 to −6.66 kcal/mol. Following the same criterion for selecting the compound with the best molecular score and interaction with the nsp10 interface, 22 compounds presented the highest binding affinities. The selection of best hits was based on the binding score of the compounds with the binding site residues. The docking score cut-off for the selection of these 22 compounds was greater than −7.0 kcal/mol. The final hits were further validated against the nsp10 interface residues by running the 22 molecules and nsp10 through the genetic algorithm by AutoDock Vina [46]. The AutoDock Vina run resulted in the 10 best hits, ranging from −8.1 to −6.8 kcal/mol (Table 1). Finally, from those 10 compounds, we analysed the best four: genkwanin-6-C-beta-glucopyranose, paraliane diterpene, citrinamide A and 4,5-di-p-trans-coumaroylquinic acid. These four compounds presented the best docking score/interaction with the interface residues and underwent further comparative interaction analysis (Figure 2). MD simulations were also used to measure the conformational dynamics of nsp10 complexes with the four compounds.

### 3.3. Binding Models of the Top Compounds with nsp10 Interface Residues

The top four compounds that demonstrated a high binding affinity and showed the most favourable interactions with the nsp10 interface residues presented in Table 1. Figure 2 demonstrates each of the compounds binding to different regions of the nsp10 interface. Table 2 lists the ADMET properties and bioactivity of the top four hits predicted from the Molinspiration Cheminformatics server. Each hit has a score between 0–5, indicating that each compound possesses considerable biological activity. Table 2 also shows the calculated Lipinski violation and experimental IC50, as well as the predicted toxicity from the pkCSM database [44].

#### 3.3.1. Binding Mode of Genkwanin-6-C-beta-glucopyranos

Genkwanin-6-C-beta-glucopyranoside is a flavonoid found in Livistona australis (Palmae), also known as cabbage tree palm, from the family Arecaceae. The antioxidant effect of genkwanin-6-C-β-glucopyranoside helped in restoring glutathione (GSH) levels in diabetic rats; GSH has a reducing capacity and protects against lipid peroxidation [60]. In vitro studies have tested the cytotoxic activity of genkwanin-6-C-β-glucopyranoside against colon, breast and liver carcinomas and have shown a high anti-proliferative activity, with IC_50_ values ranging from 0.029–0.035 µM against carcinoma cell lines. Genkwanin-6-C-beta-glucopyranoside presented a docking score of −7.2 Kcal/mol and formed interactions with four residues of the nsp10 interface: a π-cation interaction with A4424 and C4330, an H-bond formation with K4346 and van der Waal interactions with R4331 and C4330 (Figure 2A). The interaction between genkwanin-6-C-beta-glucopyranoside and the nsp10 interface occurred at residues C4330 and K4346, which correspond to the reported domain in nsp10 at residues 68–96 (PDB: 2FYG and 3R24) for the binding of short peptides K29 and TP29 [28]. These residues demonstrated the functional importance of the nsp10 interaction and the MTase activity of nsp10/nsp16 complex [28,29].

#### 3.3.2. Binding Mode of Paraliane Diterpene

Paraliane diterpene ([2S,3S,4R,5R,6R,8R,12S,13S,14R,15R]-5,8,14-triacetoxy-3-benzoyloxy-15-hydroxy-9-oxo paraliane) is a product of Euphorbia, a genus of flowering spurge plants from the family Euphorbiaceae. In traditional medicine, Euphorbia extract is used for treating warts and fistulas. Paraliane diterpene has shown anti-inflammatory and antiviral effects against human immunodeficiency virus (HIV) [61]. Experiments with MT-4 cells have shown that the antiviral activity of paraliane diterpene against HIV-1 replication arises through inhibition of the virus-induced cytopathicity and a moderate antiviral activity (EC50 = 14 mg/mL) [62]. In vivo, paraliane diterpene demonstrated comparable properties to dexamethasone [63]. In addition, it inhibits NO_2_^−^ production in a model of acute inflammation (LPS-stimulated J774 macrophages) with an IC_50_ of 0.1–10 µM [61]. Paraliane diterpene showed a docking score of −7.1 Kcal/mol and formed two H-bonds with residues N4392 and K4346 on the nsp10 interface, in addition to π-cation formation with K4346 and van der Waal interaction with C4294. The interaction residue K4346 corresponds to the previously reported nsp10 interaction domain that was targeted by K29 and TP29 short peptides [28,29].

#### 3.3.3. Binding Mode of Citrinamide A

Citrinamide A is an aromatic alkaloid isolated from endophytic fungus Penicillium citrinum from a Moroccan plant stem Ceratonia siliqua. The Penicillium genus produces a variety of bioactive compounds, such as the penicillin antibiotic. Endophytes provide several metabolites with structures that may possess biological or pharmaceutical activities. Testing citrinamide A with the antifungal medication miconazole showed that using 50 µg/mL of citrinamide A decreased the IC_50_ of miconazole from 9.1 nM to 5 nM, where it may act through inhibition of one or more of the proteins involved in infection initiation [64]. Citrinamide A formed an H-bond with K4296 and a π-sulphur interaction with C4294 from the nsp10 interface residues and showed a docking score of −7.4 Kcal/mol. AMES analysis showed no cytotoxicity, in addition to a high potency with an IC_50_ of 0.005–0.009 µM [64].

#### 3.3.4. Binding Mode of 4,5-di-p-trans-coumaroylquinic Acid

The flavonoid 4,5-di-p-trans-coumaroylquinic acid is isolated from the genus Tribulus (e.g., Tribulus terrestris) In traditional medicine, T. terrestris is used to treat conditions ranging from impotence to rheumatism, oedema, hypertension and kidney stones. The docking score of 4,5-di-p-trans-coumaroylquinic acid was −7.2 Kcal/mol and it formed four H-bonds with residues A4342, C4330, K4346 and K4348, in addition to a π-cation interaction with A4324. The interaction residues in the 6W61 structure, namely A4342, C4330, K4346 and A4324, corresponded to the interface domain of nsp10 residues 42–120 in the PDB, namely 2FYG and 3R24 that were reported to have a functional importance for stimulating nsp16 enzymatic activity [28]. In vitro studies demonstrated antioxidant activities for 4,5-di-p-trans-coumaroylquinic acid as it showed significant free radical scavenging properties with the 2,2-diphenyl-1-picrylhydrazyl radical (DPPH). This compound also appears to be a potent antioxidant, with an IC_50_ 0.039 µM [65] and a high bioactivity score of 0.43 (Table 2).

### 3.4. Conformational Dynamics and Binding Energies of nsp10 in Complex with the Selected Compounds

**Figure 3:**
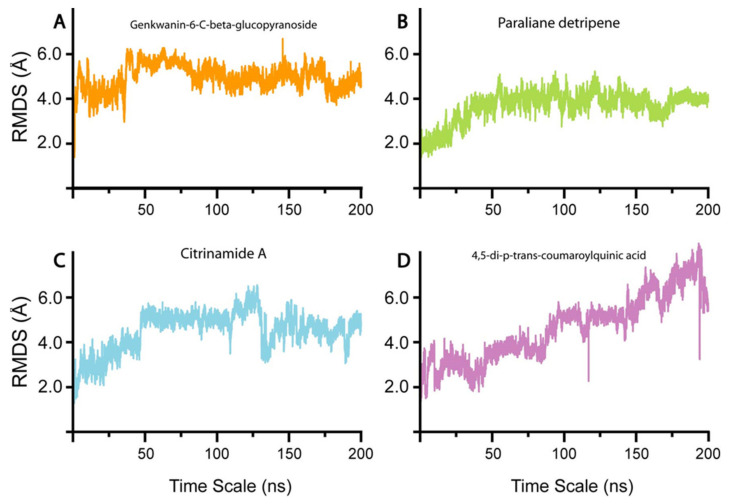
RMSD of all four system (**A**) Genkwanin-6-C-beta-glucopyranoside-nsp10 complex, (**B**) Paraliane diterpene-nsp10 complex, (**C**) Citrinamide A, (**D**) 4,5-di-p-trans-coumaroylquinic acid. The x-axis shows the time scale in ns and the y-axis the RMSD in Å.

**Figure 4:**
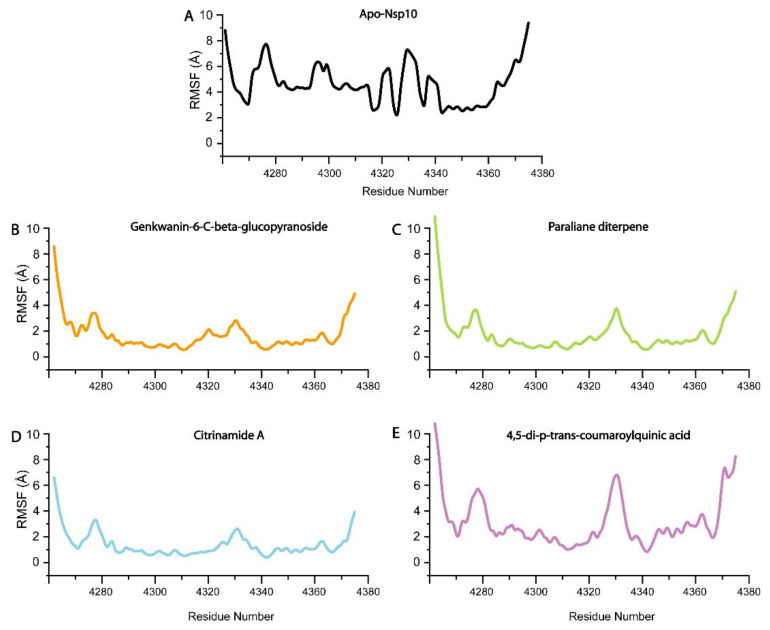
Root mean square fluctuations (RMSF) of all four system (**A**) nsp10-apo, (**B**) Genkwanin-6-C-beta-glucopyranoside-nsp10 complex, (**C**) Paraliane diterpene-nsp10 complex, (**D**) Citrinamide A, (**E**) 4,5-di-p-trans-coumaroylquinic acid. The x-axis shows the number of residues and the y-axis the RMSF in Å.

To understand the conformational stability and dynamic features of nsp10 bound to the four selected compounds, we subjected the complexes to 200 ns MD simulations (Figure 3). The RMSD trajectories of the Cα-atoms demonstrated the dynamic stability and convergence of each nsp10-compound system (Figure 3). The calculated root mean square fluctuations (RMSF) of the Cα-atoms demonstrated the residual flexibility of each compound interacting with the nsp10 interface (Figure 4). The impact of each compound on the nsp10 interface was further confirmed by applying the total binding free energy using the MMGBSA method [66], as shown in Figure 2.

#### 3.4.1. Genkwanin-6-C-beta-Glucopyronoside–nsp10 Complex

In the initial stage of the simulation from 0–6 ns, the RMSD increased to 4.0 Å (Figure 3A). The complex equilibrated and presented an average RMSD of 4.0 Å as the simulation progressed to 48 ns. Subsequently, the system converged at 50 ns, resulting in an increase in the RMSD from 4.0 to 6.0 Å. The increase in convergence of the system in the first 50 ns of the simulation indicates a highly dynamic protein-ligand complex [67]. Nevertheless, as the simulation progressed from 50 to 100 ns, the fluctuation of the system gradually decreased, resulting in a return of the RMSD to the equilibrium of 4.0 Å. As the simulation progressed to 200 ns, the system remained at equilibrium with an RMSD of 4.0 Å, with a slight convergence at 180 ns. The increase in convergence of the system at 50 to RMSD 6.0 Å indicated a highly dynamic structure, after which the system equilibrated at 100 ns to 4.0 Å, suggesting that the nsp10-genkwanin-6-C-beta-glucopyranoside complex is very stable. The apo-Nsp10 RMSF demonstrated higher conformational mobility (Figure 4A), in comparison to the RMSF of the nsp10-genkwanin-6-C-beta-glucopyranoside complex residues (Figure 4B). In general, demonstrated a stable internal dynamic between 2 and 6 Å throughout the simulations (Figure 4B). Whereas, the observed increase in residue fluctuations between amino acids 4275–4285 and 4320–4350 can be the result of the interatomic bonds between Genkwanin-6-C-beta-glucopyranoside and nsp10 residues. Genkwanin-6-C-beta-glucopyranoside forms H-bonds with I4334, D4335, H4336, G4341 and K4346. Of these, K4346 is involved in the nsp10/nsp16 interface [36]. The H-bonds are in the same vicinity as the nsp10/nsp16 interface (residues 4340–4350), resulting in a higher degree of motion. Since H-bond interactions play a crucial role in protein-ligand stability, genkwanin-6-C-beta-glucopyranoside demonstrated a strong interaction, with a free binding energy of −37.39 Kcal/mol.

#### 3.4.2. Paraliane Diterpene–nsp10 Complex

The start of the simulation showed an RMSD increase from 1–2.0 Å, followed by system convergence that showed stability until reaching an RMSD of 4.0 Å at 40 ns (Figure 3B). From 60–160 ns, the complex remained at equilibrium, with a slight convergence between 160 and 175 ns, after which the system returned to 4.0 Å. In comparison with the previous system, the nsp10-paraliane diterpene complex demonstrated a relatively more stable structure with the RMSD, reaching equilibrium during the 200 ns simulation. The RMSF showed a constant value of 2 Å throughout the 200 ns simulation, indicating a more stable nsp10 complex in comparison to apo-Nsp10 (Figure 4B). The fluctuations in the N and C terminal residues of nsp10, indicating that the interaction between nsp10 and paraliane diterpene did not affect the internal dynamics of the complex (Figure 4C). The slight fluctuations observed occur at the interface residues 4270–4290 and 4320–4350. Furthermore, the interaction of paraliane diterpene with nsp10 demonstrated a binding free energy of −33.4094 Kcal/mol. Since paraliane diterpene forms three H-bonds with nsp10 interface residues, N4293 and K4296 might have resulted in a comparatively weaker binding affinity in comparison to the nsp10-genkwanin-6-C-beta-glucopyranoside complex.

#### 3.4.3. Citrinamide A–nsp10 Complex

The nsp10-citrinamide A complex remained relatively unstable in comparison to the genkwanin-6-C-beta-glucopyranoside and paraliane diterpene complexes during the 200 ns simulation. During the initial 50 ns of the simulation, the RMSD increased from 2.0–4.0 Å. When the system equilibrated at 5.0 Å, the complex remained stable from 50 to 125 ns. Beyond 125 ns, the system started to converge, and the RMSD fluctuated until the end of the simulation (Figure 3C). This fluctuation of the system indicates that the complex is relatively unstable and requires a more extended simulation to attain stability. The RMSF fluctuations observed from residues 4290–4310 can be the result of citrinamide A forming a π-cation bond with P4290 and a π-sulphur bond with C4294 (Figure 4D). However, the increased fluctuations observed from residues 4320 to 4360 may be the result of citrinamide A forming H-bonds with residues K4296, A4355, T4358 and N4357. The strong H-bond network formed by citrinamide A with nsp10 resulted in a strong binding affinity of –29.6112 Kcal/mol. 

#### 3.4.4. 4,5-di-p-trans-coumaroylquinic acid–nsp10 Complex

The nsp10–4,5-di-p-trans-coumaroylquinic acid complex demonstrated an unstable system during the 200 ns simulation (Figure 3). From 0–50 ns, the system fluctuated between 2–4 Å, with convergence at 30 and 50 ns. Subsequently, the RMSD increased continuously to 6.0 Å during the 200 ns simulation; therefore, it never reached equilibrium, indicating an unstable system. The RMSF profile also demonstrated higher fluctuations in comparison to genkwanin-6-C-beta-glucopyranoside, paraliane diterpene and citrinamide A (Figure 4E). The increased internal fluctuations observed between residues from 4330–4360 can be due to the 4,5-di-p-trans-coumaroylquinic acid H-bond network with residues A4324 and C4330 (Figure 4E). K4348 and K4346 are also part of the nsp16–nsp10 interface residues; therefore, this H-bond network can assist in blocking the interface and result in the binding free energy of −26.3701 Kcal/mol.

## 4. Summary

In general, the top four compounds (genkwanin-6-C-beta-glucopyranoside, paraliane diterpene, 4,5-di-p-trans-coumaroylquinic acid and citrinamide A) with the highest binding affinities for nsp10 exhibited no cellular toxicity. These compounds also demonstrated bioactivity scores above 0.00, indicating significant biological activity. The Lipinski rule [68], which states that candidate compounds are more likely to be orally active, was violated by genkwanin-6-C-beta-glucopyranoside that had more than five hydrogen bond donors and by paraliane diterpene that had a molecular weight > 500 Da (Table 2). Even though these two compounds broke one of the five rules, they are likely to be orally active as they resemble other orally active drugs, such as atorvastatin and cyclosporin, that have also violated more than one of Lipinski’s rules, [69]. In our study, the four selected compounds are orally active and present high bioactivity with no toxicity [70], therefore, they represent viable compounds to block the nsp10–nsp16 interaction. 

The MD simulations and free energy binding and entropy interaction calculations also revealed which of the four compounds exhibited a more favourable interaction with nsp10. Genkwanin-6-C-beta-glucopyranoside and paraliane diterpene, in complex with nsp10, presented more stable structures than were observed with nsp10-4,5-di-p-trans-coumaroylquinic acid and nsp10-citrinamide A complexes, as depicted by the RMSD values (Figure 3). Since the formation of a stable protein-compound complex indicates a tighter binding affinity, this was further corroborated by the demonstration of binding free energies of −37.3903 ±1.3 Kcal/mol and −33.4094 ±1.3 Kcal/mol for nsp10-genkwanin-6-C-beta-glucopyranoside and nsp10-paraliane diterpene, respectively. Previous reports showed that the domain at residues 42–120 comprises an important area in the nsp10 interface [28]. For example, Ke et al. identified two short peptides, K12 (residues 68–80) and K29 (residues 68–96), from the interaction domain of nsp10 that form stable secondary structures and could significantly inhibit the 2′-O-MTase activity of nsp10–nsp16 complex in SARS-CoV [28]. In a continuation of their work, Wang et. al. reported the design of TP29, an inhibitory peptide that corresponds to residues 68–96 in the nsp10 interaction interface and that inhibited 2′-O-MTase activity in different coronaviruses in biochemical assays [29]. These results are in agreement with our findings that residues K4346 and C4330 (PDB: 6W61) appear to be important sites for the interaction between nsp10 and genkwanin-6-C-beta-glucopyranoside and paraliane diterpene that prevents nsp10–nsp16 complex formation. 

Of the four compounds, genkwanin-6-C-beta-glucopyranoside would appear to be the most favourable for further in vitro and in vivo studies. In vivo studies demonstrated that genkwanin-6-C-beta-glucopyranoside has a low IC_50_ (0.029–0.035 µM) (Table 2) indicating a good functional strength as an inhibitor of the target protein. This is further substantiated with the observation of a tenfold higher affinity to nsp10 for genkwanin-6-C-beta-glucopyranoside having than for paraliane diterpene. Even though genkwanin-6-C-beta-glucopyranoside violates one of the five Lipinski rules by having six H-bond donors, it showed a high bioactivity score of 0.48 when compared to paraliane diterpene. Additionally, with a total polar surface area (TPSA) of 170.05 Å^2^, genkwanin-6-C-beta-glucopyranoside shows a score close to the 160 Å^2^ threshold, which is a very good predictor of drug transport properties such as intestinal absorption [71]. Collectively, these properties identify genkwanin-6-C-beta-glucopyranoside as a compound with potential as a new antiviral agent for SARS-CoV-2.

## 5. Conclusions

In conclusion, we described four natural products that targeted the interaction interface of the SARS-CoV-2 nsp10–nsp16 complex. These compounds exhibited high binding affinities to nsp10 interaction interface residues and can therefore disrupt the formation of the nsp10–nsp16 complex. In particular, genkwanin-6-C-beta-glucopyranoside exhibited a stable binding, with a free binding energy of −37.3906 ± 1.3 Kcal/mol, in addition to bioactive properties that promote it as a new therapeutic target for SARS-CoV-2. Therefore, we propose genkwanin-6-C-beta-glucopyranoside as a new compound that can inhibit nsp10–nsp16 formation, thereby setting the path for functional in vitro and in vivo studies.

## Figures and Tables

**Table 1:** Docking of the top ten compounds using AutoDock Vina.

Compound Name	Vina Score
Delta-tocopherol	−8.1
1-O-linoleoyl-3-O-beta-D-galactopyranosyl-syn-glycerol	−8.2
N1,N10-di-dihydrocaffeoylspermidine	−8.0
Citrinamide A	−7.4
(9Z,12Z)-octadecadienoic acid glucoside	−7.1
Paraliane deterpine	−7.1
4,5-di-p-trans-coumaroylquinic acid	−7.2
2,3-dihydro-5,7-dihydroxy-3-((3Z,6Z,9Z,12Z,15Z)-octadeca-3,6,9,12,15-pentaenyl) chromen-4-one	−6.8
10-epi-cubebol-(alpha-xylopyranoside-triacetate)	−7.3
Genkwanin-6-C-beta-glucopyranoside	−7.2

**Table 2:** ADMET analysis, biological and toxicology properties.

Compound Name	MW.	Source	Molecule Class	Biological Activity	Lipinski Violation	AMES * Toxicity	Rat Oral LD50 (mol/kg)	Max. Tolerated Dose (Human) (log Mg/Kg/day)	IC_50_ In Vitro (µM)	T.Pyriformis Toxicity ** (log µg/L)	HBD	HBA	Rotatable Bonds No.	TPSA	Bioactivity
genkwanin-6-C-β-glucopyranoside	446	Livistona australis	Flaonoid	Antioxidant	1	-	2.77	0.405	0.029–0.035	0.284	6	10	4	170.05 Å^2^	0.41
Paraliane deterpine	598.68	Euphorbia paralias	Terpenoid	Molluscicidal	1	-	3.43	−0.308	0.1–10	0.284	1	10	9	142.50 Å^2^	0.09
Citrinamide A	431.48	Penicillium citrinum	Alkaloid	Miconazole	0	-	1.96	0.883	0.005–0.009	0.284	4	6	15	141.67 Å^2^	0.18
4,5-di-p-trans-coumaroylquinic acid	484.45	Tribulus terrstris	Phenolic	Antioxidant	0	-	2.32	0.169	0.039	0.284	5	10	9	170.82 Å^2^	0.43

* AMES toxicity test, in-vitro testing to assess the potential carcinogenic effect of chemicals. ** Tetrahymena pyriformis, the most commonly ciliated model, used for toxicological studies.

## Data Availability

Data availability upon request.
